# Aconitase Causes Iron Toxicity in *Drosophila pink1* Mutants

**DOI:** 10.1371/journal.pgen.1003478

**Published:** 2013-04-25

**Authors:** Giovanni Esposito, Melissa Vos, Sven Vilain, Jef Swerts, Jorge De Sousa Valadas, Stefanie Van Meensel, Onno Schaap, Patrik Verstreken

**Affiliations:** 1 VIB Center for the Biology of Disease, Leuven, Belgium; 2 KU Leuven, Center for Human Genetics and Leuven Research Institute for Neuroscience and Disease (LIND), Leuven, Belgium; Stanford University School of Medicine, United States of America

## Abstract

The PTEN-induced kinase 1 (PINK1) is a mitochondrial kinase, and *pink1* mutations cause early onset Parkinson's disease (PD) in humans. Loss of *pink1* in *Drosophila* leads to defects in mitochondrial function, and genetic data suggest that another PD-related gene product, Parkin, acts with *pink1* to regulate the clearance of dysfunctional mitochondria (mitophagy). Consequently, *pink1* mutants show an accumulation of morphologically abnormal mitochondria, but it is unclear if other factors are involved in *pink1* function *in vivo* and contribute to the mitochondrial morphological defects seen in specific cell types in *pink1* mutants. To explore the molecular mechanisms of *pink1* function, we performed a genetic modifier screen in *Drosophila* and identified *aconitase* (*acon*) as a dominant suppressor of *pink1*. Acon localizes to mitochondria and harbors a labile iron-sulfur [4Fe-4S] cluster that can scavenge superoxide to release hydrogen peroxide and iron that combine to produce hydroxyl radicals. Using Acon enzymatic mutants, and expression of mitoferritin that scavenges free iron, we show that [4Fe-4S] cluster inactivation, as a result of increased superoxide in *pink1* mutants, results in oxidative stress and mitochondrial swelling. We show that [4Fe-4S] inactivation acts downstream of *pink1* in a pathway that affects mitochondrial morphology, but acts independently of *parkin*. Thus our data indicate that superoxide-dependent [4Fe-4S] inactivation defines a potential pathogenic cascade that acts independent of mitophagy and links iron toxicity to mitochondrial failure in a PD–relevant model.

## Author Summary

In this work we provide mechanistic insight linking together two of the earliest observations in Parkinson's disease: the excessive build-up of iron in diseased *substantia nigra* neurons and mitochondrial dysfunction particularly increased reactive oxygen species production at the level of Complex I. We identify *aconitase* mutants as strong genetic suppressors of Parkinson-related *pink1* mutant phenotypes, both at the organismal and at the cellular/mitochondrial level. We show that the mitochondrial dysfunction in *pink1* mutants that includes Complex I dysfunction results in superoxide-dependent inactivation of the Aconitase iron-sulfur cluster, leading to the release of iron and peroxide that combine to produce hydroxyl radicals and mitochondrial failure. Consequently, scavenging free iron using expression of mitoferritin or decreasing the levels of *aconitase* both rescue *pink1* mutants; while increased wild-type Aconitase, but not a mutant that does not harbor an iron-sulfur cluster, results in severe mitochondrial defects. Given that reduced electron transport chain activity, increased oxidative stress, and natural iron build-up in the *substantia nigra* are common factors in sporadic and familial forms of Parkinson's disease, we believe that oxidative inactivation of Aconitase may represent an important pathogenic cascade underlying neuronal dysfunction in Parkinson's disease.

## Introduction

Parkinson's disease (PD) is the most frequent neurodegenerative movement disorder, but the pathways that explain disease pathology remain poorly understood [1], [2]. While the most recognized pathological feature of PD is the preferential loss of dopaminergic (DA) neurons, one of the earliest observations in *post mortem* PD brains was the accumulation of iron in the *substantia nigra* (SN) [3], [4]. Iron-mediated toxicity may thus contribute to DA neuron dysfunction but the mechanism has not been established.

Mitochondrial dysfunction is thought to be an important aspect of PD progression. Mitochondrial toxins have been linked to sporadic forms of the disease and mitochondrial defects have been described in many cell types, also in SN mitochondria of PD patients [5], [6]. Likewise some of the genetic factors linked to the disease also point to a role for mitochondria. PD-associated mutations in *pink1* and *parkin*, both affect mitochondrial function in genetic model organisms and in mammalian cells [7], [8], but how mitochondrial dysfunction and iron toxicity are linked remains elusive.

Pink1 and Parkin have been implicated in the clearance of dysfunctional mitochondria, a process dubbed mitophagy. In support, loss of *parkin* or *pink1* in different cell types in flies, results in the accumulation of swollen and clumped mitochondria [9], [10], believed to be the result of defective mitophagy [11]. Furthermore, expression of factors that promote mitochondrial fission and, as a consequence, also indirectly promote mitophagy (gain of *drp1* or loss of *opa1* or *mfn*) partially rescue defects seen in *pink1* and *parkin* mutants [12]–[14]. Further studies indicate that mitochondrial depolarization triggers the recruitment Parkin to mitochondria in a Pink1-dependent manner, facilitating mitophagy [15]. In line with this idea, over expression of Parkin in *pink1* mutants, alleviates *pink1-*associated defects [9]–[16]. Hence, Pink1 acts with Parkin to regulate mitophagy.

In parallel, *pink1* may also harbor supplementary roles. Expression of Parkin or Drp1, or loss of *opa1* or *marf* only partially rescue *pink1*-associated defects, suggesting additional pathways are contributing to the phenotype. Furthermore, loss of *pink1* function causes defects in the electron transport chain in fly and mouse cells [17], [18] that are not [19] or only partially [20] rescued by expression of Drp1. Finally, bypassing Complex I dysfunction, by expressing a yeast Complex I equivalent protein Ndi1 partially rescues the defects in *pink1* mutants, but not those seen in *parkin* mutants [19]. Hence, Pink1 may play multiple roles in mitochondria, but the relative contribution of these different pathways to the *pink1-*dependent phenotypes, including the accumulation of swollen, clumped mitochondria remains to be determined.

In an unbiased genetic screen for heterozygous suppressors of *Drosophila pink1* [21] we identified mitochondrial *aconitase* (*acon*) that encodes an enzyme catalyzing the first step of the Krebs Cycle [22]. Acon harbors an iron-sulfur [4Fe-4S] cluster [23] and we show that oxidative inactivation of this cluster in *pink1* mutants is a major cause of iron toxicity that contributes to mitochondrial swelling and clumping in *pink1* mutants. Our data are most consistent with *acon* acting downstream of pink1 and affecting mitochondrial morphology independently of *parkin*-mediated mitophagy. Thus oxidative inactivation of Aconitase is a source of iron toxicity that leads to mitochondrial defects in *pink1* mutants and we propose a model where different pathways controlled by Pink1, including mitophagy and the maintenance of ETC activity can contribute to mitochondrial failure in specific cell types.

## Results

### Aconitase downregulation suppresses *pink1* mutant phenotypes

*Pink1* mutants show a severe defect to fly caused by mitochondrial dysfunction [19], [21]. To identify genetic modifiers of *pink1*, we have tested a collection of 193 chemically induced (EMS) recessive lethal mutants that have been pre-selected for defects in mitochondrial function and neuronal communication [24]–[26], for their ability to modify the *pink1* null mutant flight defect. At the 1% significance level we isolated 5 suppressors (p<0.01) [21] and to reveal mechanisms by which the modifiers affect Pink1, we mapped one of these recessive lethal suppressors to *aconitase* (*acon*) and named it *acon^1^*. This mutant fails to complement a deletion that uncovers *acon* as well as a lethal transposon insertion in *acon* that we named *acon^2^* (Figure S1A). Sequence analysis of *acon^1^* reveals a nonsense mutation in exon 2 (Figure S1A). In addition, semi-quantitative RT-PCR and Western blot analysis indicates severely reduced mRNA and protein levels in animals that are homozygous for either *acon* allele (Figure S1C and S1D), indicating that both are loss of function alleles. Moreover we can rescue the lethality and phenotypes associated with *acon^1^/acon^2^* using a 20 kb genomic fragment encompassing the *acon* locus, yielding normal adult flies that do not show obvious behavioral abnormalities (Figure S1A, S1B). Likewise ubiquitous expression of *acon* cDNA is also able to rescue *acon^1^/acon^2^*-associated lethality (Figure S1B). Thus, one of the suppressors of *pink1* harbors a lethal lesion in *acon* and the lethality in the mutants is solely due to disruption of *acon*.

**Figure 1:**
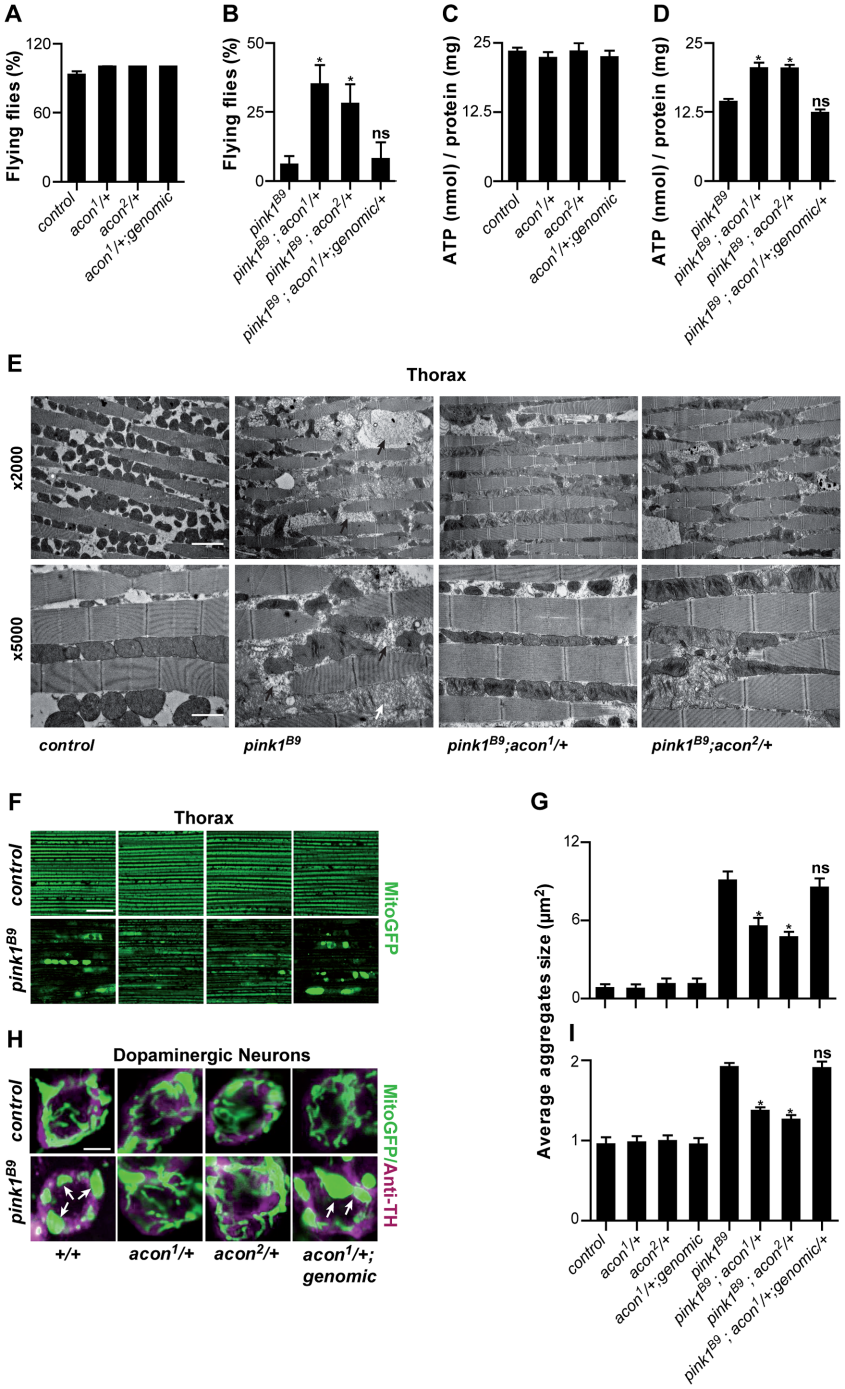
Partial loss of Acon suppresses *pink1^B9^* phenotypes. (A–D) Flight ability of 5-day-old adult flies (A, B) and ATP content in the head-thorax of 5-day-old flies (C, D). Data collected from at least 5 independent experiments. * Significantly different from *pink1^B9^*, One-way ANOVA, post hoc Dunnett p<0.01, ns: not significantly different. (E) TEM analysis of thorax. Black arrows indicate swollen mitochondria. Scale bar: ×5000 5 µm; ×2000 2 µm. (F, H) GFP-labeled mitochondria in flight muscles (F; *daGal4 UAS-mitoGFP*) and in dopaminergic (H; *pleGal4 UAS-mitoGFP*) neurons that are double-labeled with anti-tyrosine hydroxylase (magenta). White arrows indicate mitochondrial aggregates. Scale bar: muscle 10 µm; DA neurons 2.5 µm. (G, I) Quantification of average mitochondrial aggregate size. 5 images from n≥6 thoraxes and about 5 neurons per brain from n≥10 brains were analyzed. * Significantly different from *pink1^B9^*, One-way ANOVA, post hoc Dunnett p<0.01, ns not significantly different. In all panel “control” is *pink1^RV^*, a precise *P element* excision; “genomic” indicates the insertion of a construct on the third chromosome that encompasses the wild type *acon* gene, data are shown as Mean ± SEM.

Heterozygosity of *acon* significantly suppresses the flight defect associated with *pink1^B9^* mutants (Figure 1A, 1B). The extent of rescue we obtained by removing *acon*, is similar to previously reported conditions that suppress *pink1* flight defects, including adding a copy of *drp1* (*drp1^+^*) that facilitates mitochondrial fission, removing a copy of *opa1* (*opa1^S3^*), reducing mitochondrial fusion (Figure S2A, S2B), expression of Parkin, expression of yeast NDI1 that bypasses Complex I of the electron transport chain (ETC), or feeding *pink1* mutants ubiquinone or menaquinone that boost ETC function [9], [10], [13], [19], [21]. To test if the rescue that we observe is solely due to partial loss of *acon* (and not due to second site interactors on the chromosome), we determined flight but also ATP levels of *pink1* mutants with one copy of a mutant *acon* allele. While heterozygous *acon^1^* and *acon^2^* mutants alone do not show defects (Figure 1C), we find that one copy of either *acon^1^* or *acon^2^* significantly rescue the reduced ATP levels in *pink1* mutants (Figure 1D). This effect in *pink1* mutants is specific to loss of *acon* as introduction of a genomic copy encompassing wild type *acon* in *pink1^B9^;acon^2^/+* flies completely reverses both the flight and ATP level phenotypes to *pink1^B9^* mutant levels (Figure 1B and 1D). Thus, *pink1* mutant phenotypes are specifically rescued by partial loss of *acon* expression.

To further quantify the effect of *acon* on *pink1* mutant phenotypes we also analyzed mitochondrial morphology in adult indirect flight muscles using transmission electron microscopy. As previously described [9], [10], the flight muscles of *pink1* mutants exhibit swollen mitochondria with disorganized and fragmented cristae when compared to flight muscles from control flies or when compared to heterozygous *acon* mutants that do not show mitochondrial morphological defects (Figure 1E and Figure S1F). Partial loss of *acon* in *pink1* mutant results in a substantial rescue of the mitochondrial morphological defects in flight muscles, displaying substantially more intact cristae and less swollen mitochondria compared to *pink1* mutants (Figure 1E). Hence, also at the ultrastructural level, partial loss of *acon* significantly alleviates mitochondrial morphological defects in *pink1* mutant muscles.

*Pink1* mutants also show swollen and clumped mitochondria in dopaminergic neurons in the adult brain [9], [10]. To test if loss of *acon* can also rescue this defect, we expressed mitoGFP in *pink1* mutant flies and in *pink1* mutant animals heterozygous for *acon*. In line with the electron microscopy data of muscles, mitochondria in muscles labeled by mitoGFP (expressed using the ubiquitous *da*-GAL4) are spherical and aggregated in *pink1* mutants and this defect is significantly rescued by partial loss of *acon* (Figure 1F and 1G). Next, we expressed mitoGFP in dopaminergic neurons using *ple*-Gal4 (also called *TH-*Gal4). While mitochondria are organized in a tubular network in wild type dopaminergic neurons, *pink1* mutant mitochondria appear mostly as fragmented spherical aggregates in all dopaminergic neuron clusters analyzed (Figure S1H) [9], [10]. We quantified size and number of mitochondrial aggregates in the PPM3 cluster (Figure S1H and Methods). While heterozygous *acon^1^* and *acon^2^* mutants do not show defects compared to controls (Figure 1H, 1I), we find that both one copy of either *acon^1^* or *acon^2^* significantly rescue the increased size and number of mitochondrial aggregates in *pink1* mutants (Figure 1H, 1I and Figure S2A). This rescue in *pink1* mutants is specific to the partial loss of *acon* as introduction of a genomic copy encompassing wild type *acon* in *pink1^B9^*; *acon1/+* flies reverses these phenotypes back to *pink1^B9^* mutant levels (Figure 1F–1I). Furthermore, we confirm that protein levels are reduced by about 50% in *pink1^B9^*; *acon^1or2^/+* compared to *pink1* mutants and are restored in flies expressing a genomic copy of wild type *acon* (Figure S1E). Thus, together our data indicate that morphological defects of mitochondria in *pink1* mutants are significantly rescued by partial loss of *acon* expression and the mitochondrial morphological defects in *pink1* mutants are dependent on *acon* expression.

### Oxidative inactivation of [4Fe-4S] clusters results in increased H_2_O_2_ and Fe^2+^ levels in *pink1* mutants

*acon* is predicted to encode mitochondrial Aconitase (Acon), an iron sulfur cluster containing protein, that catalyzes the formation of isocitrate in the first step of the Krebs cycle [22]. To assess whether Acon localizes to mitochondria we fractionated fly tissue in cytoplasmic and mitochondrially enriched fraction and performed Western blotting using anti-Acon antibodies. Acon is enriched in the mitochondrial fraction (Figure S1G).

**Figure 2:**
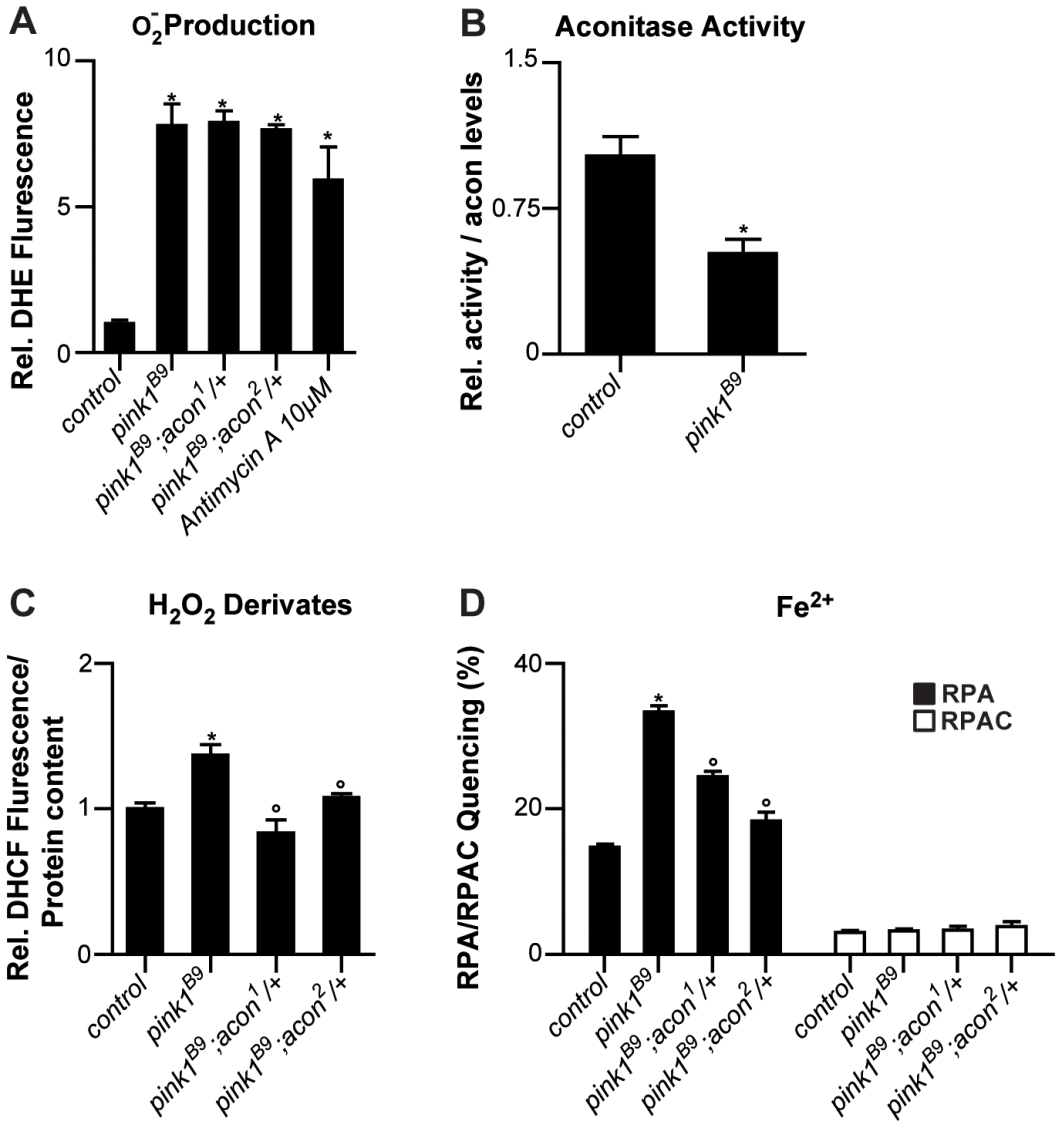
Oxidative inactivation of Acon in *pink1^B9^* mutants. (A) Superoxide production measured as fluorescence change of DHE, a superoxide sensitive dye, in isolated mitochondria from control (*pink1^RV^*), *pink1^B9^*, *pink1^B9^* mutant flies heterozygous for *acon^1 or 2^* flies as well as in *pink1^RV^* isolated mitochondria incubated with antimycin. Changes normalized to control. Data collected from 5 independent mitochondrial preparations. * Significantly different from control, One-way ANOVA, post hoc Dunnett p<0.01. (B) Relative mitochondrial Aconitase activity in *pink1^B9^* normalized to Acon protein levels. Data collected from 5 independent mitochondrial preparations. * Significantly different from control, Student's t test p<0.01. (C) H_2_O_2_ and derivates content measured as H_2_O_2_-sensitive DCHF fluorescence in fly lysates of indicated genotypes, relative to total protein content. Data collected from 5 independent mitochondrial preparations. * Significantly different from control and ° significantly different from *pink1^B9^*, One-way ANOVA, post hoc Dunnett p<0.01 (D) Fe^2+^ levels in isolated mitochondria from flies with indicated genotype. Quencing of Fe^2+^-sensitive RPA and Fe^2+^-insensitive RPAC dye are represented as percent of initial fluorescence. Data collected from 5 independent mitochondrial preparations. * Significantly different from control and ° significantly different from *pink1^B9^*, One-way ANOVA, post hoc Dunnett p<0.01. Data are shown as Mean ± SEM.

Acon harbors a single unligated iron atom in its [4Fe-4S]^2+^ cluster, and the enzyme is in this respect unique in mitochondria. Such an unligated iron atom is particularly sensitive to superoxide (O_2_^−^)-dependent oxidation [27]–[29] that results in cluster instability. Oxidation is followed by the release of Fe^2+^ and H_2_O_2_ that may contribute to oxidative damage and mitochondrial morphological defects through the formation of the potent hydroxyl radical (OH^.^) by the Fenton reaction [30]. Thus, the specific configuration of the Acon [4Fe-4S]^2+^ cluster in combination with its proximity to mitochondrially generated superoxide place Acon as a major mediator of oxidative stress in mitochondria. We therefore wondered if O_2_^−^ leaking from defective *pink1* mutant mitochondria could be a source of Acon inactivation resulting in morphological defects. To test if also in fruit flies the loss of *pink1* function results in increased O_2_^−^ production, we incubated mitochondrial preparations from *pink1* mutant flies and controls with Complex I substrates (pyruvate/malate) and used the fluorogenic probe dihydroethidium (DHE) to measure O_2_^−^ production [31], [32]. Similar to wild type mitochondria in the presence of AntimycinA, known to induce O_2_^−^ production (Figure 2A), *pink1^B9^* mitochondria show a significant increase in DHE fluorescence compared to controls (Figure 2A). These data indicate that *pink1* loss leads to increased O_2_^−^ production.

If the increased O_2_^−^ in *pink1* mutants can act via the Acon [4Fe-4S] cluster to cause mitochondrial swelling, we expect (1) that partial loss of *acon* does not rescue the increased O_2_^−^ production in *pink1* mutants; (2) that Acon enzymatic activity normalized to total Acon protein is reduced in *pink1* mutants; (3) that H_2_O_2_ and Fe^2+^ levels are increased in *pink1* mutants as a result of Acon inactivation, and (4) that this defect is rescued by partial loss of *acon*. First we assessed O_2_^−^ in *pink1* mutants heterozygous for *acon^1^* or *acon^2^* that we showed rescues mitochondrial morphological defects in *pink1^B9^*. However, in line with our model, heterozygosity for *acon* does not reduce *pink1^B9^*-induced O_2_^−^ production (Figure 2A), indicating that increased O_2_^−^ production *per se* does not induce mitochondrial morphological defects. Next we measured Acon activity in *pink1* mutant mitochondria and we find that Acon activity normalized to total Acon protein levels is significantly reduced compared to the controls. These data are in line with increased Acon inactivation in *pink1* mutants (Figure 2B), likely as a result of the increased O_2_^−^.

Further testing our model, we also measured H_2_O_2_ and Fe^2+^ content. To measure H_2_O_2_ and its radical derivatives we incubated fly lysates with the fluorescent probe dichlorofluorescein diacetate (DHCF-DA) [33]. We find a 50% increase in fluorescence in *pink1* mutant lysates compared to the control (Figure 2C). Thus, *pink1* mutants accumulate H_2_O_2_ and/or derivatives thereof. We also measured mitochondrial Fe^2+^ content by incubating mitochondrial enriched fractions with Rhodamine B-[(1,10-phenanthrolin-5-yl)aminocarbonyl]benzyl ester (RPA) [34]. In the presence of Fe^2+^, RPA fluorescence quenches and in *pink1^B9^* mitochondria, we observe a significant increase in RPA quenching compared to controls (Figure 2D). These data indicate increased mitochondrial Fe^2+^ levels in *pink1^B9^* mutants. This effect is specific, as incubating mitochondria of controls and mutants in Rhodamine B 4-[(Phenanthren-9-yl)Aminocarbonyl]benzyl ester (RPAC) that consists of the same fluorophore as RPA but without iron-chelating properties, does not show quenching in *pink1^B9^* or in controls (Figure 2D). Thus, our data indicate that *pink1^B9^* mutants harbor increased levels of Fe^2+^ and of H_2_O_2_ and/or its radical derivatives.

Next we tested if increased mitochondrial Fe^2+^ and H_2_O_2_ accumulation in *pink1* mutants is a consequence of Acon[4Fe-4S] inactivation by O_2_^−^. We therefore measured Fe^2+^ and H_2_O_2_ and its derivatives levels in mitochondria of *pink1* mutants heterozygous for *acon^1^* or *acon^2^*. While the increased O_2_^−^ production in *pink1^B9^* mutants was not reduced by heterozygous *acon*, as shown above (Figure 2A), we find that compared to *pink1^B9^*, mitochondrial Fe^2+^ and H_2_O_2_ levels are significantly lower in *pink1^B9^* heterozygous for *acon^1^* or *acon^2^* (Figure 2C and 2D). Thus, these data are consistent with the possibility that mitochondrial Fe^2+^ and H_2_O_2_ and/or its radical derivatives-accumulation in *pink1* mutants is caused by oxidative inactivation of Acon.

### Mitochondrial morphological defects are critically dependent on the Acon dose

**Figure 3:**
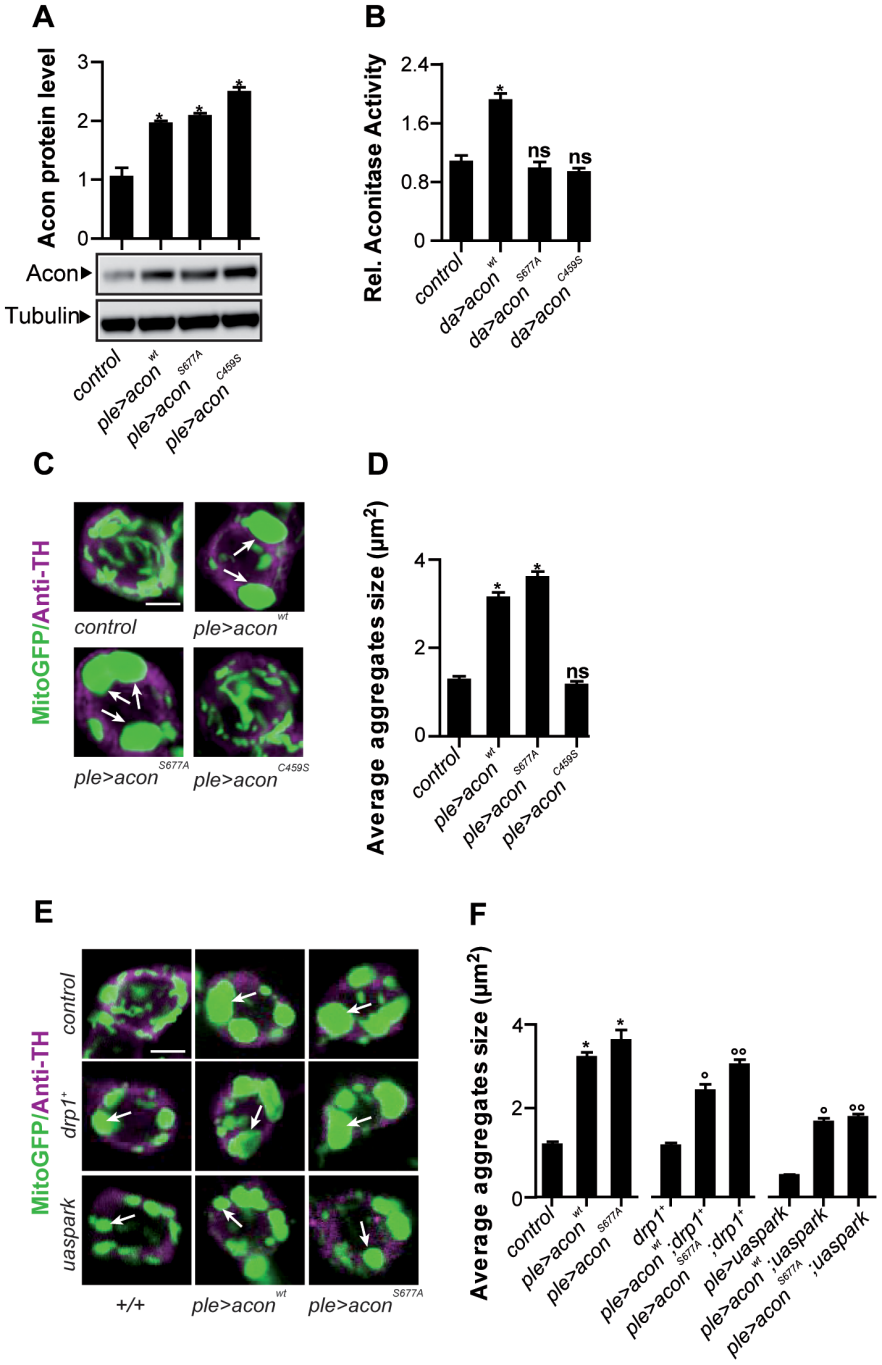
Acon[4Fe-4S] cluster induces mitochondrial defect in DA neurons that is not rescued by increased mitophagy. (A) Quantification of Western blots of fly heads expressing *UAS-acon* (see text) in DA neurons probed with anti-Acon normalized to tubulin, relative to control. Data from 3 independent experiments. (B) Mitochondrial Aconitase activity relative to the control. Data collected from 5 independent mitochondrial preparations. (C,E) GFP-labeled mitochondria in dopaminergic (DA) neurons DA neurons labeled with anti-tyrosine hydroxylase (magenta). White arrows indicate mitochondrial aggregates. Scale bar: 2.5 µm. (D,F) Quantification of average mitochondrial aggregate size. 5 neurons per brain from n≥10 brains were analyzed. The genotype of control is: *w^1118^*; *pleGal4 UAS-mitoGFP*/+ ; and of mutants that express wild type or mutant Acon is: *w^1118^*; UAS-acon^*^/+; *pleGal4 UAS-mitoGFP*/+. Significantly different * from control, ° from ple>acon^wt^, °° from ple>acon^S677A^ One-way ANOVA, post hoc Dunnett p<0.01, ns: not significantly different. Data are shown as Mean ± SEM.

Our biochemical data support a model in which oxidative inactivation of Acon and ensuing Fe^2+^ and H_2_O_2_ accumulation contributes to the mitochondrial morphology defects in *pink1* mutants. We reasoned that if partial loss of *acon* protects against mitochondrial stress in *pink1* mutants, increased levels of Acon expression may predispose cells to develop mitochondrial morphological defects, provided sufficient O_2_^−^ is around. We therefore created transgenic animals that overexpress wild type Acon (Figure 3A) resulting in increased Acon activity (Figure 3B). We then determined mitochondrial morphology using mito-GFP and the *ple*-GAL-4 driver upon expression of Acon in DA neurons. While mitochondria in DA neurons of control flies organize in a long tubular network, mitochondria in DA neurons that overexpress Acon form fragmented spherical aggregates (Figure 3C, 3D and Figure S2A). Hence, in contrast to partial loss of *acon* that rescues mitochondrial defects in *pink1* mutants, overexpression of Acon causes mitochondrial morphological defects and swelling of mitochondria in DA neurons.

Based on the finding that increased expression of Acon causes mitochondrial morphological defects we tested if pink1 mutant flies upregulate Acon expression. We measured *acon* mRNA and protein levels in *pink1* mutants, but in contrast to our expectation, we find a significant downregulation of both *acon* mRNA and Acon protein levels in *pink1* flies (Figure S2C, S2D) suggesting that an adaptive mechanism already acts in *pink1* mutants to down regulate Acon expression. Thus, the *pink1^B9^*-induced stress response results in lower Acon levels and, as shown above, further reducing Acon expression (using heterozygous *acon* mutants) is protective against mitochondrial defects in *pink1* mutants. Taken together, the data are consistent with Acon being a dosage sensitive modifier of morphological defects in mitochondria.

### Mitochondrial morphological defects as a consequence of Acon inactivation depend on its [4Fe-4S] cluster

To test if the mitochondrial morphological defects in DA neurons following Acon overexpression are induced by increased Acon catalytic activity or by the presence of an [4Fe-4S] cluster we generated transgenic flies that either overexpress a catalytic inactive Acon (Acon^S677A^) that still harbors its [4Fe-4S] cluster, or flies that overexpress an Acon without its [4Fe-4S] cluster (Acon^C459S^) and is thus also catalytically inactive [22], [35].Western blotting indeed indicates overexpression of the mutant Acon proteins (Figure 3A), and as expected, Acon enzymatic activity measured in fly head lysates is only increased when wild type Acon is expressed, and not when Acon^S677A^ or Acon^C459S^ are expressed (Figure 3B). While overexpression Acon^S677A^ in DA neurons results in obvious mitochondrial morphological defects similar to the overexpression of wild type Acon, overexpression of Acon^C459S^ is inert (Figure 3C, 3D and Figure S2A). Hence, the Acon [4Fe-4S] cluster predisposes DA neurons to mitochondrial morphological defects.

**Figure 4:**
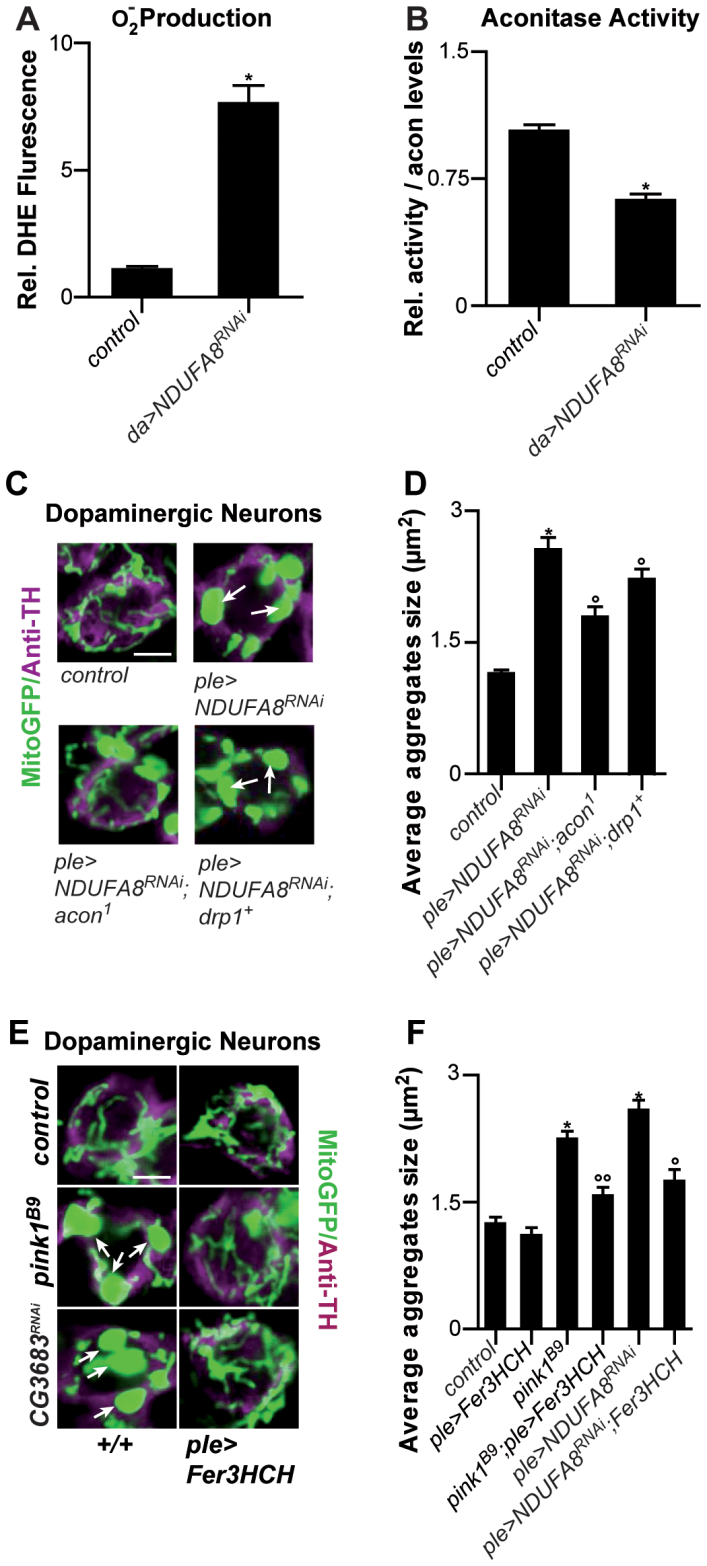
Mitochondrial morphological defects in DA neurons of Complex I–deficient and ***pink1^B9^*** **flies involve iron-mediated toxicity.** (A) Superoxide production measured as fluorescence change of DHE, a superoxide sensitive dye, in isolated mitochondria from control (*w^1118^*; *daGal4/+*) and from Complex I RNAi expressing flies (*w*; *UAS-NDUFA8^RNAi^/+*; *daGal4/+*). (B) Relative mitochondrial Aconitase activity in Complex I RNAi expressing flies normalized to Acon protein levels. Data collected from 4 independent mitochondrial preparations. * Significantly different from control, Student's t test p<0.01. (C,E) GFP-labeled mitochondria in DA neurons, labeled using anti-tyrosine hydroxylase (magenta), of control (*w^1118^*; *pleGal4 UAS-mito-GFP*/+), Complex I RNAi expressing flies (*w^1118^*; *UAS-NDUFA8^RNAi^/+*; *pleGal4 UAS-mito-GFP*/+), and such flies heterozygous for *acon^1^* (*w^1118^*; *UAS-NDUFA8^RNAi^/acon^1^*; *pleGal4 UAS-mito-GFP*/+) or overexpressing *drp1* (*w^1118^*; *UAS-NDUFA8^RNAi^/+*; *pleGal4 UAS-mito-GFP*/*drp1^+^*). In E GFP-labeled mitochondria in DA neurons *pink1^B9^*, *pink1* mutants that express mitoferritin (*pink1^B9^*; *pleGal4 UAS-mito-GFP*/*UAS-mitoFerIII*), Complex I RNAi expressing flies (*w*; *UAS-NDFUA8^RNAi^/+*; *pleGal4 UAS-mito-GFP/+*) and Complex I RNAi, mitoferritin co-expressing flies (*w*; *UAS-NDFUA8^RNAi^/+*; *pleGal4 UAS-mito-GFP/UAS-mitoFerIII*). White arrows indicate mitochondrial aggregates. Scale bar 2.5 µm. (D,F) Quantification of average mitochondrial aggregate size. Significantly different * from control ° significantly different from *ple>NDUFA8^RNAi^* °° from *pink1^B9^* One-way ANOVA, post hoc Dunnett p<0.01. Data are shown as Mean ± SEM.

Our data are in line with a model where oxidative inactivation of the Acon [4Fe-4S] cluster by O_2_^−^ contributes to mitochondrial morphological defects. To find further evidence for this idea we expressed *Drosophila* mitochondrial Ferritin (Fer3HCH) [36] in DA neurons of *pink1^B9^*, using the *ple*-GAL4 driver and assessed mitochondrial morphology using mito-GFP. We find that expression of Fer3HCH significantly rescues defects in mitochondrial morphology in *pink1^B9^* mutants (Figure 4E, Figure 2F, and Figure S2A), suggesting that iron toxicity causes mitochondrial defects in *pink1* mutants. Consistent with this model, expression of Fer3HCH in flies that over express Acon also results in a significant rescue of the mitochondrial morphological defects in the DA neurons (Figure S2E, S2F). Hence, the mitochondrial swelling as a result of Acon overexpression is at least in part mediated by iron. Together these data indicate that Acon is a critical source of Fe^2+^-mediated mitochondrial toxicity.

### Mitochondrial morphological defects upon Acon overexpression are not rescued by Drp1 or Parkin

Mitochondrial dynamics and mitophagy are critical processes in maintaining a healthy population of mitochondria. Pink1 has been implicated to regulate mitochondrial homeostasis via several mechanisms. Deregulation of these pathways may be a source of O_2_^−^, responsible for Acon inactivation. While Pink1 has been found to maintain the activity of Complex I in the ETC [17]–[20], the protein has also been linked to mitophagy in a pathway involving Drp1 and Parkin [11]–[15], [20], [37]–[40]. Dysfunctional mitochondrial parts may be segregated by the fission factor Drp1 [41], [42]. Pink1 stabilized on depolarized mitochondria then mediates Parkin recruitment causing the ubiquitination of mitochondrial proteins and activation of the autophagic machinery [41], [42]. To test if enlarged and swollen mitochondria upon Acon over expression are a consequence of defective remodeling or mitophagy we co-overexpressed Parkin, a protein that ubiquitinates mitochondrial targets, or Drp1, a mitochondrial fission factor, two conditions thought to facilitate mitophagy. While over expression of Parkin or Drp1 -as expected- result in fragmentation of mitochondria, these conditions do not rescue the defect in mitochondrial swelling and clumping induced by expression of Acon or Acon^S677A^ (Figure 3E, 3F and Figure S2A). Hence, our data suggest that the defects in mitochondrial morphology induced by Acon expression are at least in part caused independently from defects in remodeling and mitophagy.

### Mitochondiral defects caused by Complex I dysfunction are rescued by partial loss of Acon and by mitoferritin

Given that *pink1* mutants display reduced Complex I activity [17]–[20] and this feature may also be a source of increased O_2_^−^ we tested if mitochondrial swelling and clumping seen in animals where we downregulated an evolutionary conserved Complex I component, NDUFA8, can be rescued by partial loss of *acon*. First, we confirm increased O_2_^−^ production and find a concomitant inactivation of Acon activity upon RNAi-mediated downregulation of *NDUFA8* (Figure 4A and 4B). Second, we believe that this O_2_^−^ is produced at least partly independently from defects in mitochondrial remodeling because expression of Drp1 in DA neurons with reduced NDUFA8 function does not fully rescue the mitochondrial swelling and clumping phenotypes in PPM3 DA neurons (Figure 4C, 4D and Figure S2A). Next, we tested the ability of heterozygous *acon* to modulate the mitochondrial morphological defect induced by *NDUFA8* RNAi and find that heterozygous *acon* is more effective than expression of Drp1 in rescuing the mitochondrial deficits in DA neurons (Figure 4C, 4D and Figure S2A). Likewise, and in line with our model, expression of mitoferritin (Fer3HCH) also alleviates mitochondrial defects in animals that express RNAi to *NDUFA8* in DA neurons (Figure 4E, 4F and Figure S2A). Hence, our data suggest that Acon is inactivated by ETC-derived O_2_^−^ causing oxidative stress.

**Figure 5:**
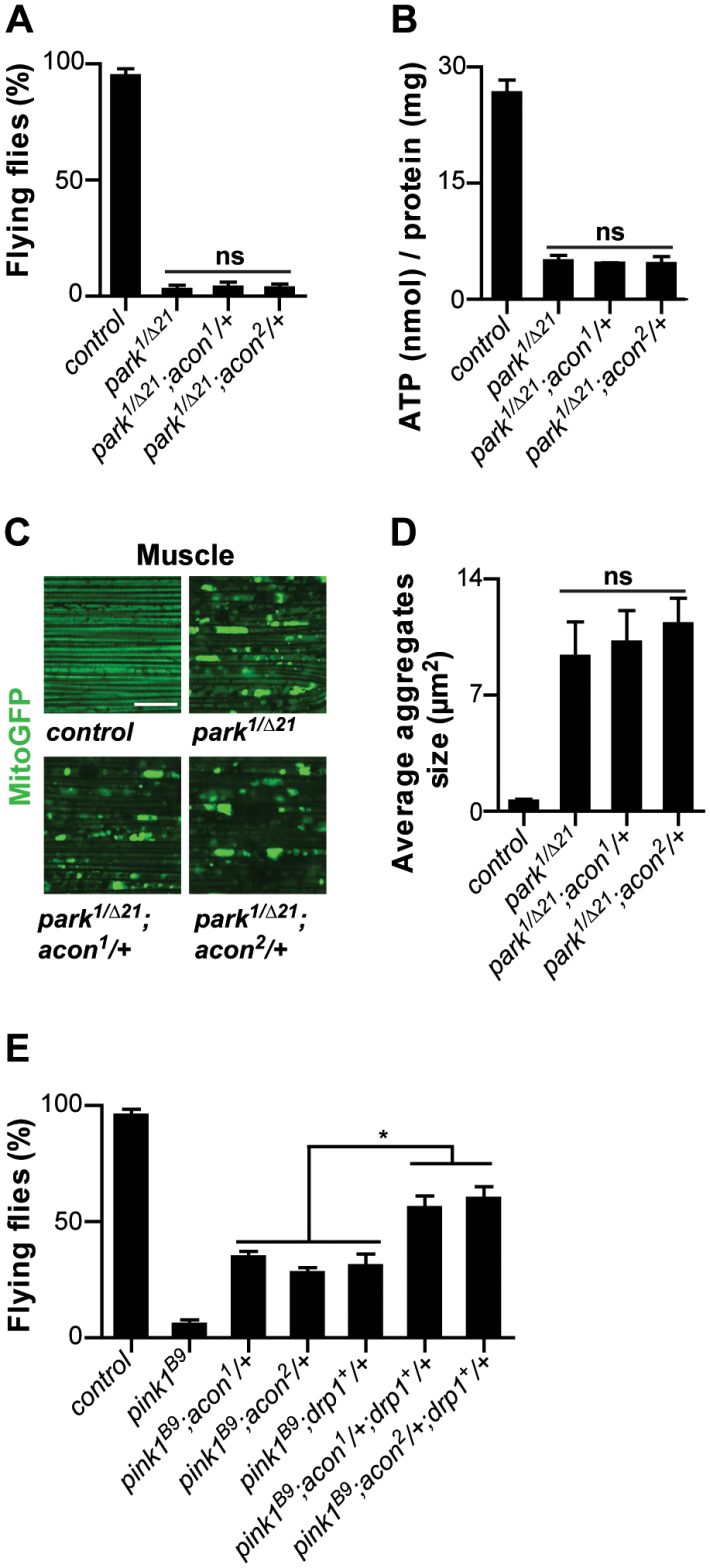
Acon inactivation and Parkin-mediated mitophagy act in parallel in *pink1* mutants. Analysis of *parkin* mutants (*pakin^1/Δ21^*) and parkin flies heterozygous for *acon^1or2^* (A) Flight ability of 5-day-old adult flies and (B) ATP content in the head-thorax of 5-day-old flies. Data collected from at least 5 independent experiments. (C) GFP-labeled mitochondria in flight muscles (*daGal4 UAS-mitoGFP*). Scale bar: muscle 10 µm. (D) Quantification of average mitochondrial aggregate size. 5 images from n≥6 thoraxes. (E) Flight ability of control flies, *pink1^B9^*, *pink1^B9^* heterozygous for *acon^1or2^* heterozygous for *acon^1or2^* or overexpressing *drp1* and *pink1* mutants with a combination of *acon^1or2^* heterozygousity and *drp1* overexpression. * Significantly different from *pink1^B9^*, One-way ANOVA, post hoc Dunnett p<0.01, ns: not significantly different.

Our work suggests that mitochondrial morphological defects in *pink1* mutant DA cells can be of different origin: both O_2_^−^-dependent Acon inactivation or loss of Parkin-dependent mitophagy yield swollen and clumped mitochondria. Alleviating the defects induced by either pathway using heterozygous *acon* or expressing Drp1 or Parkin both rescue the mitochondrial morphological defects in *pink1* mutants (this work; [12]–[14], [16]). To further support this notion, we first assessed if mitochondrial defects in *parkin* mutants can be rescued by partially removing *acon* function. *parkin* mutants display enlarged and swollen mitochondria in muscles and DA neurons, many of the flies also fail to fly and animals harbor lower ATP levels. In contrast to removing *acon* function in *pink1* mutants, heterozygosity for *acon* fails to rescue the inability of *parkin* mutants to fly, their reduced ATP levels and their defects in mitochondrial morphology (Figure 5A–5D). Hence, our data suggest that *acon* acts independently from defects in Parkin-dependent mitophagy. Finally if our model is correct, we reasoned that the combination of Drp1 expression and *acon* heterozygosity in *pink1* mutants should yield additive ‘super rescue’. We therefore tested the ability of these flies to fly and find that they fly significantly better than *pink1* mutants or than *pink1* mutants partially rescued by either Drp1 expression or by heterozygous *acon* (Figure 5E). Hence, these data are in line with Pink1 controlling different mitochondrial pathways that can be targeted largely independently. We speculate that increased O_2_^−^ derived from a defective Complex I in *pink1* mutants is an important contributor to Acon inactivation, but other sources of O_2_^−^ may contribute to mitochondrial failing as well.

## Discussion

**Figure 6:**
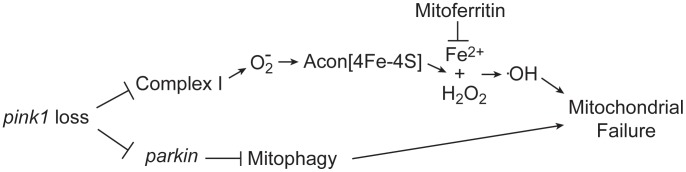
Model of oxidative Acon inactivation in *pink1* mutants. Pink1 loss induces increased superoxide production that inactivates the Acon[4Fe-4S] resulting in the generation of Fe^2+^ and H_2_O_2_. These combine to form hydroxyl radicals that lead to mitochondrial failure. Mitoferritin chelates Fe^2+^ and is thus able to rescue mitochondrial failure in *pink1* mutants. Acon inactivation act in parallel to Parkin-mediated mitophagy in controlling mitochondrial integrity.

Iron accumulation in the *substantia nigra*, systemic mitochondrial dysfunction and oxidative stress have all been implicated in PD pathology; however, a link between these factors remains elusive. Here we show that oxidative inactivation of Acon generates iron-mediated oxidative stress that contributes to mitochondrial swelling in *Drosophila pink1* mutants (Figure 6). Inactivation of Acon[4Fe-4S] clusters could contribute to mediating O_2_^−^ toxicity by simultaneous release of Fe^2+^ and H_2_O_2_ [43] that combine in the Fenton reaction to generate highly toxic hydroxyl radicals [30], [44] (Figure 6). Hydroxyl radicals can induce mitochondrial permeability transition and swelling [45]–[47], in line with electron microscopic analyses of *pink1* mutants where mitochondria appear swollen and show disorganized cristae [9], [10] (Figure 1). Four major findings support that this iron-mediated toxic mechanism is an additional important aspect of mitochondrial dysfunction in *pink1* mutants. First, we find increased O_2_^−^ production, increased Acon inactivation and more Fe^2+^ and H_2_O_2_ accumulation in *pink1* mutants (Figure 2). Second, partial loss of Acon reduces Fe^2+^ and H_2_O_2_ accumulation and alleviates *pink1-*associated phenotypes including mitochondrial morphological defects in muscle and DA neurons (Figure 1). Third, overexpression of wild type Acon in dopaminergic neurons produces a mitochondrial morphological defect and this effect is completely dependent on the presence of the [4Fe-4S] cluster in Acon (Figure 3). These data also indicate mitochondrial integrity is sensitive to Acon [4Fe-4S] cluster dosage. Finally, chelating iron by expressing mitochondrial Ferritin is sufficient to rescue *pink1* mitochondrial morphological defects (Figure 4). Thus, our data suggest that inactivation of Acon and iron accumulation might be a pathogenic mechanism triggered by loss of *pink1* and increased superoxide, linking iron accumulation and mitochondrial failure.

Acon inactivation is dependent on O_2_^−^ that, amongst other sources (see below), may be produced in defective mitochondria. While various mitochondrial insults can result in increased O_2_^−^ production, our work is most consistent with Parkin-dependent mitophagy being not the major source of Acon inactivation in *pink1* mutants. The mitochondrial morphological defects induced by Acon overexpression were not strongly rescued by expressing Drp1, a condition that indirectly promotes mitophagy and *parkin* mutants were not majorly rescued by partial loss of *acon* (Figure 3 and Figure 5). In contrast, mitochondrial morphological defects in DA neurons of flies with reduced Complex I activity are significantly rescued when *acon* is heterozygous (Figure 4). Hence, Acon seems to act in a Pink1-dependent pathway that can operate largely independently of mitophagy (Figure 6).

Defects at the level of Complex I are often associated with increased leaking of the toxic O_2_^−^ [48], [49], and likewise, systemic inhibition of Complex I mimics features of PD in animal models [50]–[53]. Previous work in flies or mice has indicated reduced ETC function [17]–[20] in *pink1* mutants, and we show that this condition results in mitochondrial morphological defects in an Acon-dependent manner. Similar to *pink1* mutants, RNAi-mediated knock down of an evolutionary conserved Complex I component, *NDUFA8*, also results in an increased production of superoxide as well as in Acon inactivation. We show that these biochemical changes correlate with mitochondrial morphology defects in dopaminergic neurons that can be rescued by partial loss of *acon* or by over-expression of mitoferritin that scavenges the released Fe^2+^ [36] (Figure 4).

It is interesting to note that increased O_2_^−^ production *per se* is not sufficient to generate mitochondrial morphological defects, and that the presence of sufficient amounts of Acon is required. Indeed, our data indicate that *pink1* mutants heterozygous for *acon* show increased levels of O_2_^−^ but normal mitochondrial morphology. Our data also indicate that upstream events in *pink1* mutants that result in increased O_2_^−^ production contribute to mitochondrial morphological defects because of oxidative inactivation of Acon. In line with this, overexpression of the mitochondrial superoxide dismutase 2 (SOD2) that scavenges O_2_^−^, successfully rescues mitochondrial swelling phenotype of *pink1* in DA neurons [54]. Given that both genetic forms of PD as well as sporadic cases of PD show ETC defects [5], [6], [17]–[19], our work may be relevant for idiopathic cases that suffer from mitochondrial dysfunction as well. Acon inactivation and iron-mediated toxicity might thus have a more general role in the pathogenesis of PD.

While *pink1* loss affects numerous cell types, our data also start to provide insight as to why DA neurons in the *substantia nigra* are more vulnerable in PD. While overexpression of Acon or downregulation of Complex I produces mitochondrial morphological defects in DA neurons, in *Drosophila* flight muscles mitochondria appear morphologically largely normal (data not shown). These data suggest a tissue-specific response in that Acon inactivation has a stronger impact in DA neurons than in muscle cells. Each cell type is exposed to various sources of O_2_^−^, but DA neurons in particular are exposed to dopamine-induced oxidative stress that is a source of O_2_^−^ [55]–[57]. Furthermore, the *substantia nigra* in humans is naturally rich in iron [58] and this feature may lower the threshold for hydroxyl radical production in the Fenton reaction that is facilitated by Acon inactivation. *Pink1* mutations or environmental factors in some sporadic cases of PD already result in increased levels of O_2_^−^, but we hypothesize that in DA neurons, additional dopamine-induced oxidative stress may facilitate Acon inactivation and hydroxyl radical production providing insight into one of the pathways underlying mitochondrial failure in *pink1* mutants.

## Methods

### *Drosophila* stocks and maintenance

Flies were raised on standard cornmeal and molasses medium at 25°C. *w^1118^*; *UAS-mitoGFP*, *w^1118^*; *daGal*, *w1118*; *pleGal4*, *w*; *UAS-4EBP* and *w^1118^*; *Mi{ET1}Acon^MB09176^/SM6a* (*acon^2^*) and were obtained from Bloomington stock center (Indiana, USA). *w^1118^ pink1^B9^* and *w^1118^ pink1^RV^*, *parkin^1^* and *parkin^RV^* [59] were provided by Jongkyeong Chung (Advanced Institute of Science and Technology, Korea) [10]. *parkin^Δ21^* mutant flies were a gift from Graeme Mardon (Baylor College of Medicine) [60] and *drp1^+^* genomic rescue constructs were provided by Hugo Bellen (Baylor College of Medicine) [61] *w^1118^*; *UAS-Fer3HCH* was provided by Dr Fanis Missirlis (Qeen Mary University of London, UK). *w^1118^*; *UAS-CG3683^RNAi^* (*w^1118^*; *P{GD16787}v46799/CyO*) was from the Vienna *Drosophila* RNAi Center (VDRC) [62].

### Molecular biology and biochemistry

The genomic clone CH322-18I04 was obtained from BACPAC Resources (Children's Hospital Oakland).

*UAS-Acon^wt^* was generated by PCR amplification of BDGP cDNA clone LD24561 using primers: AconcDNA.F (5′ ATGGCTGCGAGATTGATGAACG) and AconcDNA.F (5′ TTACTGGGCCAGCTCCTTCATGC). The S677A and C459S mutations were introduced in the primers and the mutated cDNAs were generated by overlap extension PCR using the following primers: S677A.F (5′GAcgCACCCTCGCCGTAGTTCTCATC) S677A.R (5′AACTACGGCGAGGGTGcgTC), C459.F (5′GGTCCCtCcATTGGACAGTGGGATCG) and C459.R (5′CGATCCCACTGTCCAATgGaGGGACC). All constructs were cloned into the EcoRI and NotI restriction sites of pUAST-attB [63]. Following sequencing, transgenic flies were created at GenetiVision Inc. (Houston, USA) using PhiC31 mediated transgenesis in the VK1 docking site (2R, 59D3) [64].

For quantitative RT-PCR, total RNA was isolated using TRI Reagent (Sigma-Aldrich) according to the manufacturer's protocol. Subsequently, the RNA samples were cleaned up using the RNeasy Mini Kit with the on-column DNAse treatment (Qiagen). 1 µg of total RNA was used as a template for synthesis of oligodT-primed double stranded cDNA using the SuperScriptIII First-Strand Synthesis System (Invitrogen). 20 ng cDNA of each sample was used for *acon* SYBR Green PCR Master mix (Applied Biosystems) and the following primers were used: aconRT-F (5′ TCGTGCCATTATCGTCAAGTC) and aconRT-F (5′ AGGTTGAGCAGGGAGATTTTG). All experiments were performed in triplicate and run on a Roche LC480 system. The data were normalized utilizing RP-49, a ribosomal gene, using following primers: RP-49-F (5′ ATCGGTTACGGATCGAACAA) and RP-49-R (5′ GACAATCTCCTTGCGCTTCT).

For Western blots, flies were homogenized in cold T-PER buffer (ThermoScientific) with complete protease inhibitor mixture (Roche). Protein concentration was determined by BCA protein quantification kit (Pierce). Samples were diluted in 2-mercaptoethanol 10% SDS loading buffer and boiled for 5 min and 15 µg of proteins were separeted on pre-cast 4–12% NuPage Bis-Tris gels (Invitrogen). Following transfer to nitrocellulose, blots were probed with primary antibodies: 1∶5000 Anti-ACO2 (AbGent), 1∶1000 anti-Tubulin (B5–12, Sigma) and 1∶1000 HRP coupled secondary antibodies (Jackson immunolabs). Blots were developed with Western-Lightning-ECL (PerkinElmer) and imaged. Quantification was performed using gel analyzer tool in ImageJ software from the US National Institute of Health (http://rsb.info.nih.gov/ij/).

### Flight assay

Batches of 5 days old male flies were transferred to an empty vial (5 cm D, 10 cm H). Flies were allowed to climb above a marked line at 9 cm height; the vial was gently tapped and visually scored for flying flies. Flies at the bottom were removed and the remaining flies were retested. Flies that fly twice were assigned a score of 1, the others a score of 0.

### ATP measurements

ATP content was determined as described [10]. 5 days-old flies with abdomen dissected out were homogenized in 50 µl of 6 M guanidine-HCl 100 mM Tris and 4 mM, EDTA, pH 7.8. These homogenates were snap-frozen in liquid nitrogen and then boiled for 3 min. Samples were then centrifuged and the supernatant was diluted (1/50) in extraction buffer, mixed with luminescent solution (ATP Determination Kit, Invitrogen) and luminescence was measured on an EnVision Multilabel Reader (Perkin Elmer). ATP (nmol) was determined using a standard curve and normalized to protein content (mg) measured by BCA assay (Pierce).

### Muscle section and TEM

Thoraxes were fixed in paraformaldehyde/glutaraldehyde, postfixed in osmium tetroxide, dehydrated and embedded in Epon. Sections 80 nm thick were stained with uranyl acetate and lead citrate and subjected to TEM analysis.

### H_2_O_2_ levels

H_2_O_2_ was measured as described [33]. 4–5 adult flies were homogenized in 50 µl cold lyses buffer T-PER (Thermo scientific) and the homogenate were cleared by centrifugation at 1000×g for 5 min at 4°C. 140 µL of PBS containing 50 µM of DCFH-DA (molecular probe) were added to 10 µL of lysate in a 96-well plate format and incubated at 25°C for 10 minutes in the dark. DCFH-DA fluorescence (485exc/530em) was measured using Wallac Victor^2^ 1420 (Perkin Elmer). Fluorescence intensity was normalized to the protein amount (BCA, Pierce) and expressed as relative to the control.

### Mitochondria isolation

Fifty flies were gently crushed in 1 ml chilled mitochondrial isolation medium (Mitosciences) by using a porcelain mortar and pestle, then spun twice at 1,000×*g* for 5 min at 4°C to remove debris. The supernatant was then spun at 12,000×*g*, for 15 min at 4°C. The pellet, containing the mitochondria, was washed with 1 ml of isolation medium and resuspended in 40 µl of isolation medium supplemented with complete protease inhibitor mixture without EDTA (Roche).

### Superoxide production

Mitochondrial Superoxide production was measured as described [32]. 10 µg of mitochondria were incubated in experimental buffer (EB: 125 mM KCl, 10 mM Tris-MOPS, 1 mM KPi, 10 µM EGTA-Tris, pH 7.4, 25°C) supplemented with 1.25 mM Pyruvate/1.25 mM malate and 5 µM DHE (Molecular probe) in a 96-well plate format for 10 min. The fluorescence was measured (485exc/590em) using Wallac Victor^2^ 1420 (Perkin Elmer). Fluorescence intensity was normalized to the initial value and expressed as relative to the control. 10 µM antimycin A was used to induce superoxide production in control mitochondria.

### Fe^2+^ measurements

For mitochondrial ferrous iron level measurements, 10 µg of mitochondria were resuspended in isolation buffer (Mitosciences) and incubated with 20 µM of RPA or RPAC (Squarix Biotechnology) in a 96-well plate format at room temperature for 10 min. RPA/RAPC fluorescence (560 exc/600 em) was measured using Wallac Victor^2^ 1420 (Perkin Elmer). Quenching was calculated as percent of initial fluorescence.

### Aconitase activity

Aconitase enzyme activity microplate kit (Mitosciences) was used according to the manufacturer's protocol to measure Aconitase activity. 20 µg of mitochondria were incubated with assay buffer and the activity was measured by following conversion of isocitrate to cis-aconitate as in increased in 240 nm UV absorbance. Measurements were recorded over 30 min. at 1 min intervals and aconitase activity were calculated from the linear increase in absorbance and normalized to the amount of aconitase, determined by western blot, in the same mitochondrial preparation. Values were reported as relative activity to the control.

### Mitochondrial morphology in DA neurons

Brain dissection and whole-mount immunohistochemistry for tyrosine hydroxylase (TH) was performed as described [65]. Primary 1∶100 antibody against TH (Chemicon) and secondary alexa 555 (Invitrogen) were used. Brains were imaged on a Zeiss LSM 510 META confocal microscope using a 63xoil NA 1.4 lens. Mitochondrial tagged GFP (mito-GFP) was visualized using 488 nm laser and 500–530 band pass emission filter. Because mitochondrial morphology is sensitive to environmental conditions, variations did occur from batch to batch. We only compared flies of different genotypes if normal mitochondrial morphology was observed in the control samples (Figure S1H–S1H″) in the same batch. For quantification of mitochondrial aggregates size and numbers, DA neurons of PPM3 cluster (Figure S1H–S1H″) were scored. Quantification of aggregate size was done using “analyzing particles” plugin in ImageJ (http://rsb.info.nih.gov/ij/): rounded particles were automatically detected and the average surface area of aggregates in each neuron was determined as total area occupied by aggregates/number of aggregates.

### Mitochondrial morphology in flight muscles

Adult flies were fixed in PBS with 5% formaldehyde and 0.4% Triton for 3 hours. Thoraxes were dissected in PBS and mounted in vectashield (Vector Laboratories) and were imaged on a Zeiss LSM 510 META confocal microscope using a 63xoil NA 1.4 lens. Mitochondrial tagged GFP (mito-GFP) was visualized using 488 nm laser and 500–530 band pass emission filter. For muscle section with same area were scored and quantification of mitochondrial aggregates was performed as described above.

## Supporting Information

Figure S1 (A) Schematic representation of the *acon* gene. C→A in *acon^1^* is cytosine adenine transition that results in a STOP codon; The insertion site of Mi{ET1}Acon^MB09176^ is indicated. (B) complementation test table of different heteroallelic combinations, “genomic” indicates a genomic fragment containing the wild type *acon* locus and da>uasAcon indicates flies with ubiquitous expression of qcon cDNA; “−” means fail to complement, and “+” means adult fertile flies emerge. (C) Quantification of *acon* mRNA by semi-quantitative RT-PCR in embryos. (D) Quantification of Acon protein levels in embryos normalized for tubulin levels using Western blotting. The presence of remaining Acon protein in *acon^1 or 2^* mutants may indicate maternal component. control is *w^1118^* and data were collected from 5 independent samples. (E) Quantification of *acon* protein levels by Western blot in 5-day-old adult flies, anti-Acon normalized to tubulin, relative to control. Data collected from at least 4 independent experiments. * Significantly different from *pink1^B9^*, Student's t test p<0.01. (F) TEM analysis of thorax. Black arrows indicate swollen mitochondria. Scale bar: ×5000 5 µm; ×2000 2 µm. (G) Western blot analysis on mitochondrial and cytoplasmic fractions using antibodies against Acon and ComplexV. (H) DA neuron clusters in the protocerebrum of the *Drosophila* brain with identified clusters indicated. (H′) Magnification of the PPM3 cluster and (H″) of a single PPM3 neuron. Scale bar: 50 µm (H) 5 µm (H′) 2.5 µm (H″).
(TIF)

Figure S2 (A) Quantification of the mitochondrial aggregate number per DA neurons in PPM3 cluster. Data collected from 5 neurons per brain in at least 10 brains. Significantly different * from control, ° from *pink1^B9^*, °° *ple>NDUFA8^RNAi^*, ns not significantly different. One-way ANOVA, post hoc Dunnett p<0.01. Data are shown as Mean ± SEM. (B) Flight ability of *pink1^B9^*, *pink1* mutants overexpressing *drp1* (*pink1^B9^;drp1^+^*) or with reduced *opa1* gene dosage (*pink1^B9^*;*opa^S3^*). * Significantly different from *pink1^B9^*. (C) Quantification of *acon* mRNA by semi-quantitative RT-PCR in 5 day-old controls (*pink1^RV^*) and in *pink1^B9^* flies. (D) Quantification of Acon protein levels in 5-day-old control and *pink1^B9^* mutant flies using Western blotting with anti-Acon and normalized for tubulin levels, relative to control. Data were collected from 5 independent experiments. * Significantly different from control, Student's t test p<0.01. (E, F) Quantification of the mean number of mitochondrial aggregates per DA neuron and of average mitochondrial aggregate size of GFP-labeled mitochondria in controls (*w^1118^*; *pleGal4 UAS-mitoGFP*/+) in flies over expressing wild type Acon in DA neurons (*w^1118^*; *UAS-acon^wt^/+*; *pleGal4 UAS-mitoGFP*/+) and in flies overexpressing wild type Acon and mitoferritin in DA neurons (*w^1118^*; *UAS-acon^wt^/+*; *pleGal4 UAS-mitoGFP*/*UAS-mitoFerIII*). Significantly different * from *ple>acon^wt^ t*-test: p<0.01. In all panels data are shown as Mean ± SEM.
(TIF)
