# Supplementary figures and images for: Aconitase Causes Iron Toxicity in Drosophila pink1 Mutants
Source: PLoS Genet. 2013 Apr 25;9(4):e1003478. doi: 10.1371/journal.pgen.1003478 (PMC3636082; doi:10.1371/journal.pgen.1003478)

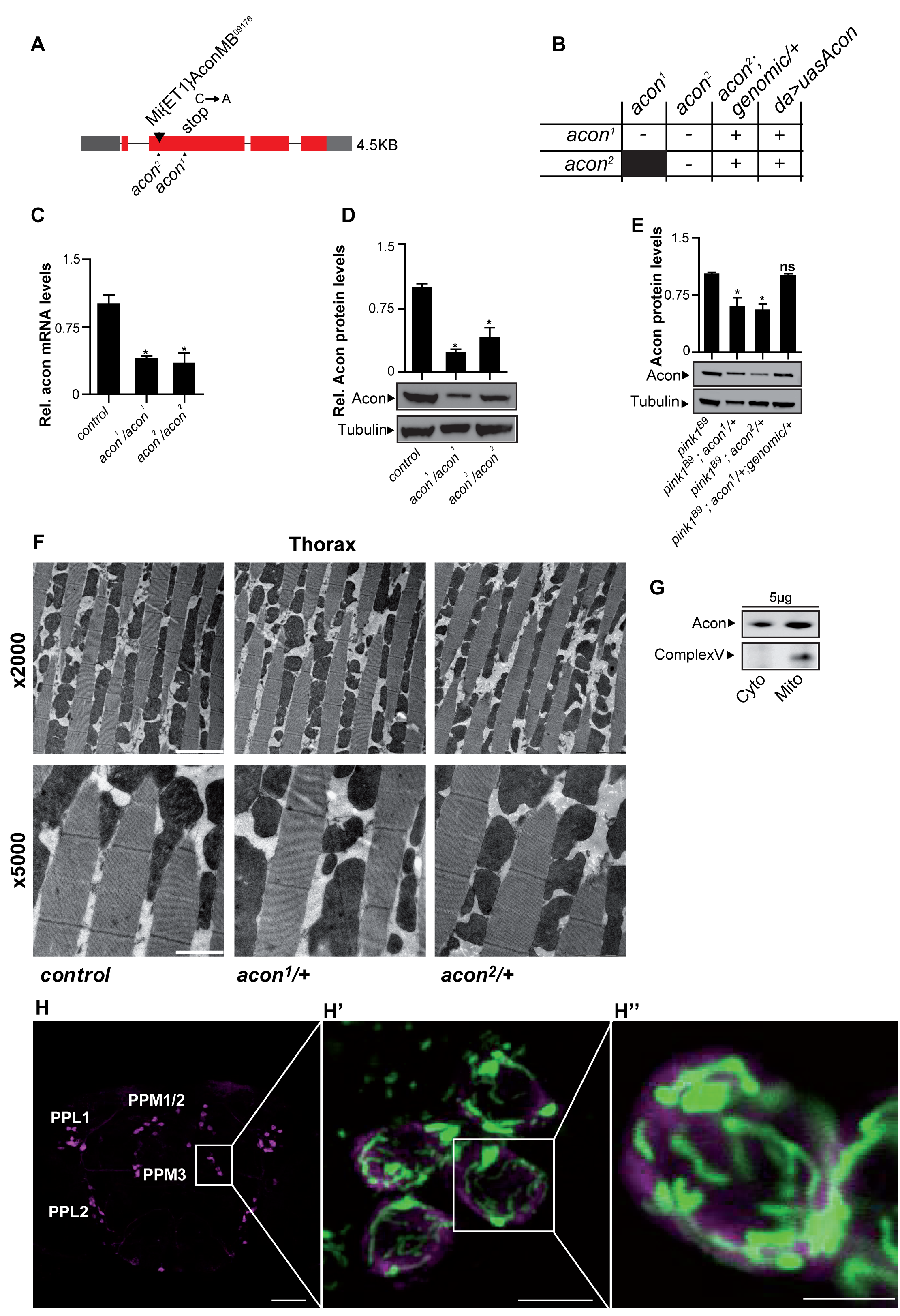

Supplement: Figure S1 — (A) Schematic representation of the acon gene. C→A in acon1 is cytosine adenine transition that results in a STOP codon; The insertion site of Mi{ET1}AconMB09176 is indicated. (B) complementation test table of different heteroallelic combinations, “genomic” indicates a genomic fragment containing the wild type acon locus and da>uasAcon indicates flies with ubiquitous expression of qcon cDNA; “−” means fail to complement, and “+” means adult fertile flies emerge. (C) Quantification of acon mRNA by semi-quantitative RT-PCR in embryos. (D) Quantification of Acon protein levels in embryos normalized for tubulin levels using Western blotting. The presence of remaining Acon protein in acon1 or 2 mutants may indicate maternal component. control is w1118 and data were collected from 5 independent samples. (E) Quantification of acon protein levels by Western blot in 5-day-old adult flies, anti-Acon normalized to tubulin, relative to control. Data collected from at least 4 independent experiments. * Significantly different from pink1B9, Student's t test p<0.01. (F) TEM analysis of thorax. Black arrows indicate swollen mitochondria. Scale bar: ×5000 5 µm; ×2000 2 µm. (G) Western blot analysis on mitochondrial and cytoplasmic fractions using antibodies against Acon and ComplexV. (H) DA neuron clusters in the protocerebrum of the Drosophila brain with identified clusters indicated. (H′) Magnification of the PPM3 cluster and (H″) of a single PPM3 neuron. Scale bar: 50 µm (H) 5 µm (H′) 2.5 µm (H″). (TIF) [file pgen.1003478.s001.tif]

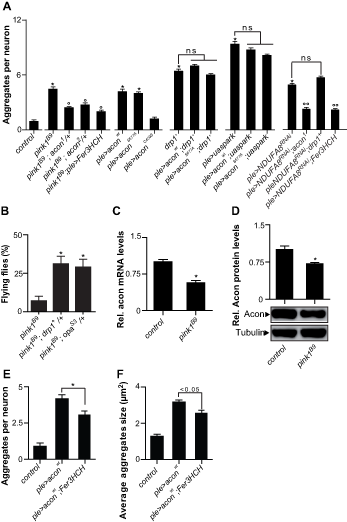

Supplement: Figure S2 — (A) Quantification of the mitochondrial aggregate number per DA neurons in PPM3 cluster. Data collected from 5 neurons per brain in at least 10 brains. Significantly different * from control, ° from pink1B9, °° ple>NDUFA8RNAi, ns not significantly different. One-way ANOVA, post hoc Dunnett p<0.01. Data are shown as Mean ± SEM. (B) Flight ability of pink1B9, pink1 mutants overexpressing drp1 (pink1B9;drp1+) or with reduced opa1 gene dosage (pink1B9;opaS3). * Significantly different from pink1B9. (C) Quantification of acon mRNA by semi-quantitative RT-PCR in 5 day-old controls (pink1RV) and in pink1B9 flies. (D) Quantification of Acon protein levels in 5-day-old control and pink1B9 mutant flies using Western blotting with anti-Acon and normalized for tubulin levels, relative to control. Data were collected from 5 independent experiments. * Significantly different from control, Student's t test p<0.01. (E, F) Quantification of the mean number of mitochondrial aggregates per DA neuron and of average mitochondrial aggregate size of GFP-labeled mitochondria in controls (w1118; pleGal4 UAS-mitoGFP/+) in flies over expressing wild type Acon in DA neurons (w1118; UAS-aconwt/+; pleGal4 UAS-mitoGFP/+) and in flies overexpressing wild type Acon and mitoferritin in DA neurons (w1118; UAS-aconwt/+; pleGal4 UAS-mitoGFP/UAS-mitoFerIII). Significantly different * from ple>aconwt t-test: p<0.01. In all panels data are shown as Mean ± SEM. (TIF) [file pgen.1003478.s002.tif]
